# Why Do People Like Loud Sound? A Qualitative Study

**DOI:** 10.3390/ijerph14080908

**Published:** 2017-08-11

**Authors:** David Welch, Guy Fremaux

**Affiliations:** 1 School of Population Health, University of Auckland, Private Bag 92019, Auckland 1142, New Zealand; 2 Triton Hearing, Whangerei 0110, New Zealand; guy.fremaux@tritonhearing.co.nz

**Keywords:** loud music, noise-induced hearing loss, ecological model

## Abstract

Many people choose to expose themselves to potentially dangerous sounds such as loud music, either via speakers, personal audio systems, or at clubs. The Conditioning, Adaptation and Acculturation to Loud Music (CAALM) Model has proposed a theoretical basis for this behaviour. To compare the model to data, we interviewed a group of people who were either regular nightclub-goers or who controlled the sound levels in nightclubs (bar managers, musicians, DJs, and sound engineers) about loud sound. Results showed four main themes relating to the enjoyment of loud sound: arousal/excitement, facilitation of socialisation, masking of both external sound and unwanted thoughts, and an emphasis and enhancement of personal identity. Furthermore, an interesting incidental finding was that sound levels appeared to increase gradually over the course of the evening until they plateaued at approximately 97 dBA Leq around midnight. Consideration of the data generated by the analysis revealed a complex of influential factors that support people in wanting exposure to loud sound. Findings were considered in terms of the CAALM Model and could be explained in terms of its principles. From a health promotion perspective, the Social Ecological Model was applied to consider how the themes identified might influence behaviour. They were shown to influence people on multiple levels, providing a powerful system which health promotion approaches struggle to address.

## 1. Introduction

Loud sound causes damage to the auditory system, and is regarded as annoying with serious impacts on physical and mental health [1]. On the other hand, it is present in many activities that people do for enjoyment: fitness centres [2], sports events [3], personal audio systems [4], live music events [5], bars [3] and nightclubs [6] have all been shown to have high levels of sound. On the face of it, this appears to be a contradiction. Why would people enjoy a stimulus which causes discomfort and negatively impacts health? Data reflecting the attitudes of young adults who enjoy loud music in nightclubs may provide a better understanding of this. Furthermore, interpretation of these data from a theoretical perspective is important to allow future research to apply the findings. These are the aims of this research.

Questionnaire-based findings suggest that young people’s attitudes towards loud music are mixed and depend on what they perceive to be normal as well as their own intrapersonal factors such as personality and symptoms of noise exposure such as tinnitus and fear of hearing loss [7,8,9]. On the other hand, one conclusion of this type of research is the need to look beyond the individual to societal level influences.

A major source of leisure-noise exposure for young people is nightclubs [6]. They may account for around 70% of the total leisure noise exposure in youth [10]. Clubbers experienced, on average, an equivalent continuous noise level of close to 98 dBA over their average attendance time of five hours a week [11]. When comparing this with the maximum noise exposure acceptable for a working life of 42 years, 10 years clubbing would generate more than 60% of the acceptable noise exposure for that working lifetime [11]. Bar managers and DJs may use music to retain customers, and even control the crowd and reduce conflict [12,13]. Loud sound may have other positive effects on business: when music is loud, men drink more, and more quickly, which may be explained by either the high sound level leading to higher arousal or reducing social interaction [14,15,16]. On the other hand, some young adults have been reported to find the sound levels at nightclubs too high [17,18,19]. The social importance of loud music has been demonstrated by its use at events where it is desirable to draw people together and generate a sense of common feeling, such as political rallies [20]. Parallels may exist with the risks of smoking tobacco [21] and alcoholic drinks [22], which have both been shown to cause harm to people and yet are associated with enjoyment and are socially acceptable. 

The Social Ecological Model provides a useful way of considering the factors that influence health related behaviours [23]. The advantage of applying the Social Ecological Model to health-risk behaviours is that, while it accounts for individual attitudes and beliefs, it also takes into consideration the impacts of higher-level aspects of the social environment, an approach which has been called for by previous research in the area [8]. According to the model, a person’s decisions about health are based on levels of influence: the intrapersonal level refers to the person’s own thoughts and attitudes; the interpersonal level is the direct influence of other people with whom one associates; the community level refers to the cultural influences on health behaviours; and the policy level refers to the influence of laws and other aspects of government policy.

On the intrapersonal level, personality would be expected to influence the appreciation of loud sounds: they provide intense stimulation and arousal, and therefore may be sought out by people high in the trait of ‘sensation seeking’ [24]. The intrapersonal level would also include factors such as personal preference for style and genre of music as well as a desire for rebelliousness [25]. The interpersonal level refers to the influence of sound on interactions with others: listening to music in groups, either informally or at concerts is common, and this may partly reflect a desire for group membership with others who adopt similar styles and tastes [26]. The community level will reflect the accepted practices around loud music, such as when it is played and how loud it sounds. The policy level is governed by legal requirements for noise levels in the workplace [27], which do not appear to be enforced in environments such as nightclubs or concerts. The Social Ecological Model predicts a lack of good health behaviour when influences towards health are not present at all levels of the model. 

In previous, theoretical work, we proposed the Conditioning, Adaptation and Acculturation to Loud Music (CAALM) Model [28]. This was based on three processes: (1) an initial physiological adaptation that enables people to overcome the discomfort associated with loud music; (2) a classically conditioned response whereby the repeated pairing of loudness with perceived benefits of loud music itself (e.g., masking, social benefits, arousal, excitement) and other frequently associated benefits in bars and clubs (e.g., dancing, fun, friends, alcohol, other drugs); and (3) an acculturation process wherein large groups of people (who have undergone the conditioning phase of the model) begin to perceive loud music as the norm and to associate it with the cultural expression of celebration and fun. The CAALM Model may inform efforts to reduce exposure to loud sound, particularly in leisure settings, and an appreciation of the role of the three processes could provide targets for interventions.

Understanding more clearly why people like loud sounds may hold the key to the design of more effective hearing conservation programmes. The personal rewards of loud sound are immediate, whereas it may be years before the costs are experienced [29]. Improved understanding of the motivations towards dangerous noise exposure may help in developing influences that would have positive impact on the intrapersonal, interpersonal and cultural levels of the social ecology. The current study investigated why people report liking loud sounds. We focussed particularly on exposures to amplified music in bars and nightclubs, because of the high rates of exposure through this source and its strong association with enjoyment of the loudness of the sound. Participants included both clubbers—consumers of the loud sound—and those who control the sound levels within clubs such as managers and DJs. The latter group were interviewed, in part, to address the question posed by previous research [17,18,19], why is music often played at levels that many people find too loud? We conducted the research via semi-structured interviews (i.e., with open-ended questioning) to allow participants to express their own thoughts about their appreciation of loud sound, in their own way [30,31]. We hoped that this might provide richer and more original than might have been obtained from a quantitative questionnaire. The findings were compared to the components of the CAALM Model and the Social Ecological Model was used as a means of placing the themes that emerged from the interviews into perspective.

## 2. Materials and Methods

In a preliminary phase, we recorded sound levels in clubs in Auckland City, New Zealand using a calibrated CEL-350 dBadge Casella dosimeter positioned as close to the ear as possible, at the lapel or collar. Care was taken to make sure the dosimeter did not attract attention. Dosimeters were calibrated to 114 dBA prior to every measurement taking place. The dosimeter logged sound level measures (Leq dBA) every minute. Measurements were taken across four different clubs beginning at 9:15 p.m. and ending at 2:00 a.m. and made by one of the authors (Guy Fremaux) on Friday and/or Saturday nights across three weeks. Sampling was ad hoc, and the exercise was not intended to provide a rigorous description of Auckland nightclubs, but rather to see whether the sound levels in clubs in Auckland were consistent with the levels reported elsewhere [3].

The main part of the research consisted of interviews with people who were involved in leisure noise exposure, either as customers or as those who set the sound levels.

### 2.1. Participants

Sixteen people were recruited by advertisement and interviewed: eight 18–25 year olds (five female and three male) who regularly attend nightclubs, six musicians/DJs/sound engineers (two female and four male), and two bar managers (both female). We wished to obtain the views of people who knew and enjoyed loud sound in order that the themes expressed would relate to the reasons why people appear to *want* to expose themselves to it, despite the physical danger it poses.

### 2.2. Data Collection and Analysis

Semi-structured interviews of approximately one hour’s duration were conducted by author Guy Fremaux, recorded on an audio device and transcribed by the same author. Semi-structured interviews allow participants to frame their thoughts in their own words and to deviate from the interviewer’s topics as they see fit, allowing greater scope for personal interpretation of the topic than would a list of questions. Five topics were introduced by the interviewer: why people liked loud sounds (all groups); quiet and loud recreational activities (all groups); views on loudness at recreational venues (all groups); management of sound levels (musicians/DJs/sound engineers and managers only); and the role of sound from a business perspective (managers only). Analysis of the data around sound management and the business perspective was conducted separately from the other data. 

The number interviewed was driven by the concept of saturation: an initial set of participants were interviewed and the themes raised identified; if these were repetitive, no more interviews need be conducted and where new themes are generated, further participants were recruited on the basis that saturation had not yet been achieved. In interviewing 16 participants, we were comfortable that we had achieved reasonable saturation, though of course it is possible that further themes may emerge with more interviews. 

Each interview was read through repeatedly until common responses were identified and a two-stage coding approach was used, where initial codes were applied and then a secondary process of collapsing and re-coding was done. This exercise was conducted across the two authors. A key principle of qualitative research is to extract clarity from the complexity present in natural text or speech. The task is to structure and present the data in terms of themes which allow a reader to develop an understanding of the area based on originality and commonality of themes. Common responses to each theme were grouped and representative quotes used to support the findings [32]. This approach allows themes to emerge from the data without applying a theoretical model at the analytical stage.

The study had approval from the University of Auckland’s Human Participants Ethics Committee (Ref. 9756) and informed consent was obtained from participants. 

## 3. Results

We have presented the results in two sections: sound levels and interview findings. Though the interviews were primarily aimed at understanding why people enjoy loud music, the interviews sometimes led in other directions and participants commented on loud sound more generally. These comments have been included in that they shed light on the themes presented.

### 3.1. Sound Levels

**Figure 1:**
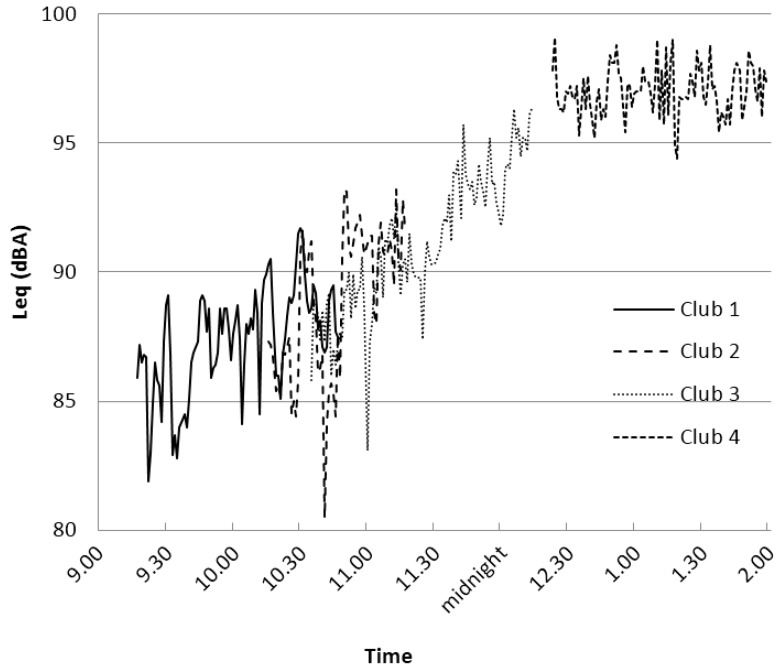
Music level (dBA Leq) per minute measured in four different Auckland clubs.

To establish comparability with other places, sound levels were measured in a set of representative Auckland clubs. Music in the Auckland clubs reached average continuous levels of around 97 dBA. Interestingly, the sound levels at different clubs appeared to follow a similar trend during the early part of the night, increasing steadily to a maximum of around 97 dBA Leq from around midnight (Figure 1).

### 3.2. Why Do People Like Loud Sound?

**Figure 2:**
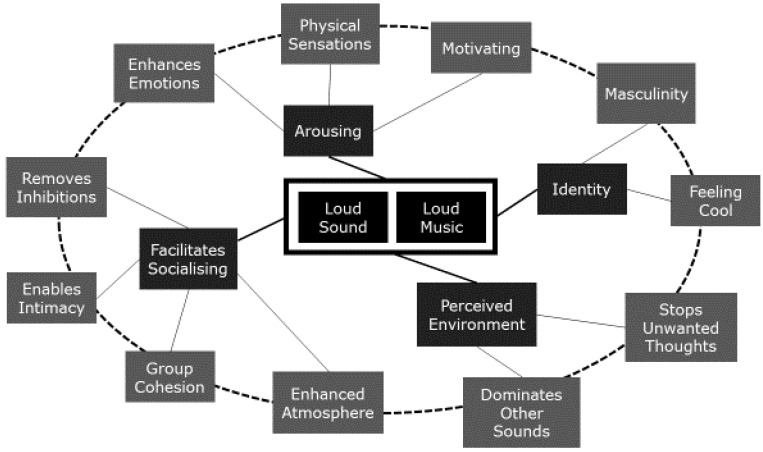
Mindmap of themes about the enjoyment of loud sound. In the people interviewed, loud sound was often loud music, and while other sources of loud sound were mentioned, most themes were associated with music. Four main themes were identified, and each contained sub-themes. In the diagram, the dotted ring represents the intuitive links between the sub-themes and demonstrates how they may support each other.

When participants were interviewed about why they liked loud sounds in general, including, but not limited to, music, four main themes emerged (Figure 2). These were: that loud sound is arousing, that it enables greater socialisation, that it masks unpleasant things, and that it emphasises personal identity.

#### 3.2.1. Theme 1: Arousal

Arousal is an enhanced physiological state where the body and senses are alert and active and emotions intensified. It reflects activity in fundamental parts of the nervous system, and is associated with responses to loud sounds due to brainstem mechanisms and associated sympathetic activity and increased wakefulness. According to the CAALM Model, people who have been conditioned to loud sound would also experience these effects due to conditioning. Consistent with this were three main subthemes: enhancing emotions, motivation, and providing direct physical sensations

##### Enhancing Emotions

Loud sounds were linked to causing a positive emotional state. Respondents felt loud sounds they liked made them feel: ‘positive’, ‘happier’, ‘upbeat’, ‘enthusiastic’, ‘energized’ and even ‘alive’. Loud sounds, particularly music, made people feel that their emotional states were intensified, presumably due to the heightened sense of arousal.

“They make me feel happy and energized and I want to turn it up even louder.”

It was also believed that loud sounds make others feel good, especially if the sounds had meaning to the person or an association with good memories.

“Like petrol heads, they associate it with their hobbies and what makes them feel good.”

In line with the CAALM concept of classically conditioned responses to loudness in music, it was reported that hearing loud sounds previously associated with positive experiences, would trigger the same positive feelings.

“It translates into fun and happy memories. Every time you hear that sound again, it conjures up those feelings again … it can change your mood wherever you are.”

Furthermore, the loudness of music was thought to enhance emotions that were already present in the piece of music.

“(I think they like it) to enhance how they feel. It sort of enhances the emotions that the music gives out—whether it’s angry or positive—when it’s loud.”

Thus, by providing the music with greater power to deliver emotional activity, loudness may be used as a means of applying greater musical control over one’s emotional state. The heightened arousal associated with the loudness creating, albeit vicariously, greater emotive valence. In keeping with this, loud sounds/music were also seen as a way of removing unwanted emotions.

“You can transfer your stress into the sound, or music, and that lets it go.”

So emotional arousal may occur, both directly, as a result of the loud music, and indirectly as a result of a learned association between the loud sound and previous positive emotional experiences.

##### Motivation

Another side of arousal is a physical tendency to move or act; a sense of potential. Participants reported that loud music was able to help people get going, especially for physical exercise or dance.

“It just helps you get into what you are doing.”

*“Especially when exercising … if you feel you want to stop, it keeps you motivated.”*

“The loud beat makes you want to move your body and be more active.”

This ability of loud sounds to motivate or encourage people to move was sometimes linked to adrenaline, in the colloquial sense of an internal sense of arousal, consistent with the CAALM Model concept of loud music stimulating the reticular activating system.

“It (loud sound) gets your adrenaline pumping.”

##### Physical Sensation

Activation of the balance organs and sensory organs in the viscera by loud sound has been observed and one theme that emerged in these interviews appeared to reflect this. The physical feeling of loud sounds vibrating through the body was pleasurable.

“I like the feeling of music going through my body ….”

#### 3.2.2. Theme 2: Facilitates Socialising

According to the CAALM Model, loud music would have impacts on a social level. It would provide a context for social interactions with a wide group of friends, establish a social milieu in which to interact positively with strangers, and also mask intimate social interactions to provide a degree of privacy in a crowded environment. In the interviews, the ability of loud sounds to positively influence social experiences had four sub-themes: improving the social atmosphere, group cohesion, removing inhibitions, and enabling intimacy.

##### Improving the Social Atmosphere

Loud music, was associated with drinking alcohol, a popular concomitant of social activity in New Zealand. It was felt that loud music encouraged drinking as a part of social situations with friends. The implication of this may be that loud music would produce a state of social cohesion akin to the effects of social drinking.

“Loud music puts you in the mood to drink when you’re with your mates.”

In this context, the classically conditioned response predicted by the CAALM Model was again expressed. It was felt that other people enjoy loud sounds, simply because of the conditioning of ‘fun’ to loud music.

“I think maybe they like it because it’s fun, and it’s associated with fun.”

The word ‘fun’ is often used to describe loud music, and especially in the social context. For a stimulus such as loud music to take on an emotional valence such as this, a learned association appears to be necessary.

##### Group Cohesion

The role of loud music as a component of a culture is consistent with the concept of ‘acculturation’ within the CAALM Model. When loud music is present, those who like it perceive it to be an integral part of an experience on a level consistent with a cultural norm. Loud music was associated with forming a connection with others, a sense of belonging to the group through the sharing of an experience.

“Everyone wants to be part of an experience, especially when it’s music, and share it with each other. Loud music makes you feel as one.”

“They feel included when it’s loud.”

“The sound, the same movie or song, builds a common rapport.”

##### Removing Inhibitions

Consistent with the parallels between loud music and social drinking described above, the music was believed to reduce social inhibitions. This also relates to other themes of privacy and intimacy described below. Participants felt that loud sounds facilitated social interaction by removing people’s social inhibitions.

“Loud sounds break down people’s inhibitions, because of what is going on around them it gives them more anonymity.”

“They (loud sounds) make you let loose and socialise.”

##### Enabling Intimacy

In a related but different theme, loud sounds were also seen to facilitate “intimate” (presumably physical) social interactions, particularly in nightclubs, because they made conversations more difficult.

“Loud music in clubs past midnight gives them an excuse not to talk to people and instead be intimate.”

In terms of the CAALM Model, the subthemes within Facilitating Socialising may be seen as conditioned effects: responses to repeated pairings of loud sound (often music) with other social activities. Thus, from the perspective of a person conditioned to enjoy loud sound, a cloud of other associations, especially social, may be experienced.

#### 3.2.3. Theme 3: Perceived Environment

A third theme that emerged in the data was that loud sounds were liked because they provided an escape for people by masking aspects of their minds (thoughts) or the world (other sounds) that were unpleasant or unwanted. The ability of loud sounds to focus attention by simultaneously ‘drawing people in’ as well as blocking out other sounds was credited for its ability to distract.

##### Replacing and Masking Thoughts

Loud sounds, particularly music, were seen as a form of ‘escapism’ to distract people from their thoughts and feelings. In particular, the feeling of ‘losing oneself in the music’ was felt to be facilitated by loudness. In terms of the CAALM Model, this theme represents an ‘internal’ benefit of loud sound: a direct effect of loud sound making it harder to think—about anything—which is perceived as beneficial when negative thoughts are intrusive on a person’s life.

“It’s just escapism. You lose yourself in the music. It makes you forget about other things, everyday problems and stuff.”

“They are a distraction from thoughts and feelings.”

##### Distracting and Masking Other Sounds

The other side of the benefits of loud sound proposed by the CAALM Model is that of ‘external’ benefits: ways in which loud sound influences (and is seen to improve) a person’s experiences of the external world. A benefit of loud music was seen as the blocking out of other unwanted sounds in the environment.

“They also distract you from other sounds going on around you.”

What is more, loud music gave people a sense of control of their soundscape or ‘personal aural space’.

“It blocks out life and noise.”

The CAALM Model description of the direct benefits of loud sound divides them into internal (relative to a person’s mind) and external. Based on the subthemes reported here, the loudness of sound creates masking effects which cross this divide, but which are related to thoughts separately from other sounds.

#### 3.2.4. Theme 4: Emphasises Identity

While participants were not willing to apply this aspect of loud sound to themselves, they felt that others may like loud sounds for this reason. In particular, it was felt to emphasise a masculine identity and ‘being cool’. We presume that responses about masculinity related to culturally-accepted norms for the genders, with masculinity being associated with activity and danger versus femininity which tends to be associated with calmness and tranquillity. It should also be borne in mind when considering this theme that responses were often elicited by ‘indirect’ questions such as, “Why do you think other people might enjoy loud sounds?” In questioning people about sensitive beliefs, this type of question may allow people to express ideas that they would not express if asked directly about their own feelings.

##### Masculinity

Participants thought that some people may like loud sounds because they associated them with masculinity, and used them to emphasise their masculinity. Particular reference was made to car enthusiasts and loud engine noises.

“With car enthusiasts, it’s like ‘Who is the manliest?’… They’re trying to be manly.”

##### Being ‘Cool’

It was also believed that loud sounds were associated with a ‘cool’ image:

“My brother likes listening to loud music because he thinks he is cool.”

*“Using a loud chainsaw to cut down a tree, you feel pretty good about yourself. Same with cars, if you rev them up, you feel like a bad-arse.”*

The ironically-acronymmed CAALM Model includes a neurophysiological component based on the known fear and autonomic activation responses to loud sound in mammals. It proposes that part of the attraction of loud sound is the thrill that people gain from controlling the natural fear response. Based on the themes presented here, this may go further and represent a positive benefit of a sense of toughness or ability to control and master one’s fear. Though the term ‘manliness’ was used, the ability to overcome a fear-response, a sense of self-control and power, may be pleasurable for both genders.

#### 3.2.5. Summary of Themes about Why People Like Loud Sound

People felt that loud sounds worked on several levels. On a physiological level, loud music aroused and excited them. On a social level, loud music would work both to draw people together in a group, and to isolate people intimately, despite crowded environments. At the level of the perceived environment, loud music could both shield a person from their own unwelcome thoughts, and from unwelcome intrusion by outside noise. Loud sounds could even make people feel a stronger identity, particularly of power and toughness. Put together, these concepts would make an environment filled with loud music appealing in many ways. The findings were broadly consistent with the CAALM Model interpretation, and also realised the concepts and extended it.

### 3.3. Views on the Level of Music

As may be expected, the positive perceptions associated with loud music are reflected in the ways that nightclubs wish to market themselves. The views on loud music showed both similarity and differences between those who were attending for fun and those for whom it was a business.

The bar manager participants felt that loud music was a requirement, that people would expect loud music, in line with the CAALM conceptualization of a culture of loud sound.

“It (music) needs to be loud at nightclubs, everyone expects it and it’s what they go for.”

They felt that the loudness had several major benefits: 

#### 3.3.1. Venue Promotion

The transmission of loud music into the street informs people about the presence of entertainment and thus serves to draw them in.

“Loud sound shows the club, bar, or even party, is on and the doors are open.”

#### 3.3.2. Masking

This theme was about setting the music to provide a masking effect allowing for privacy, in line with the themes expressed above.

“The idea is to make it so that other people can’t hear intimate conversations. So that you can have a lot of people in close proximity, but it is still private.”

#### 3.3.3. Fun

Music was seen as key to create an atmosphere which would seem attractive patrons. This perception on the part of those in control of venue music levels fitted with the concept of a loud music culture in the CAALM Model.

“At the same time it creates an exciting atmosphere.”

“Loud sounds bring more people in. It’s what people look for and you need to create the atmosphere for people to dance and “socialise” in the way they do nowadays.”

#### 3.3.4. Increasing Music Levels

Bar managers described the acceptance of loud music by themselves and their staff. They also appeared to follow a system for setting the level of music, in line with the observations shown in Figure 1.

“The volume gets louder at night and the lights go down.”

“Some places I’ve worked at, bars and clubs, they just turn the music up at particular times regardless of how many people are there. That’s their policy.”

The increase in sound level of music at venues as it gets later at night is also linked to the effects of patrons drinking and conversing more loudly.

“Also, people get louder when they get drunk, so sound levels need to go up during the night.”

Furthermore, there was a sense that the loudness would increase in response to the crowd.

“The loudness is dictated by the situation, the more the people drink, the more comfortable they feel, the more inhibitions they lose, the more they want the music to be turned up loud. The band then reacts to this and becomes louder.”

#### 3.3.5. Attitudes of Clubbers

On one hand, there was agreement with the idea that clubs should be loud places from the ‘consumer’ participants (clubbers).

“Live music is never too loud.”

“I like loud music … because it is exhilarating but you are in a safe environment.”

On the other hand, people sometimes found it too loud.

“You know it’s going to be loud, but still sometimes it is too loud ….”

“Sometimes at nightclubs you can’t hear what people are saying, and it’s hard to even order a drink. What’s the point of going out and being out with a group of friends when you can’t hear each other?”

“Pubs are generally too loud. I want to be able to talk to others but the music is too loud for that.”

This latter point differentiated between clubs, where loud music was appropriate, and ‘pubs’, more traditional environments which often play music at high levels but which do not tend to encourage dancing or other aspects of clubs and are seen as places to converse as well as drink and eat.

It was felt that people yelling in an attempt to communicate above the music was responsible for uncomfortable loudness.

“If it is too loud at clubs, I think it is the people. I think the people get louder with the music. They are in close contact to you, trying to compete with the sound/noise level. People yelling in your ear is more of a problem than the music.”

Interestingly, participants who attended nightclubs believed that they would still attend if the music was not as loud:

“If it were a little quieter at clubs I’d still go, and I think I’d like it more.”

“The loudness is part of the environment, but it doesn’t define the experience. It’s more important that you’re there with your friends … things like the quality of the music are important too.”

#### 3.3.6. Summary of Views on the Level of Music in Clubs

People enjoy loud music, and leisure venues serve to provide this enjoyment. Those who run nightclubs appear to view loud music as a necessity, both from the point of view of providing a good service to their patrons, and from doing good business themselves. This is in line with the CAALM Model concept of acculturation around loud sound, and suggests that clubs cater to this in similar ways to the provision of alcoholic drinks and lighting effects to create an atmosphere that will attract their clientele. Interestingly, people who attend nightclubs reported that they sometimes find them too loud. This may reflect satiation to the conditioned effects of loud sound, and might represent an avenue for amelioration of the dangerous levels of noise that many people are exposed to during leisure activities.

## 4. Discussion

In line with predictions based on the CAALM Model [28], loud sound produced a number of desired outcomes: it arouses and excites; it both draws people together socially and separates them to allow intimacy in crowded environments; it can mask and replace unwelcome thoughts; it can mask unwanted sound and provides a new environment in its place; and it gives people an identity of coolness and toughness. Clubs have high-level sound (Figure 1), and there is a desire and expectation from customers and staff for this, however there appeared to be disparity in that customers reported that music was sometimes too loud for their taste, consistent with previous findings [17]. 

Consideration of the data suggested that these underlying themes could themselves be linked into a self-perpetuating system that would support and drive a person to feel better about themselves, their social circle, and the environment generally (Figure 2). The ring around the outer themes shown in Figure 2 suggests how this system might lead to a strong influence on behaviour. For instance, with an arbitrary starting point at the physical sensations associated with arousal, and moving (again arbitrarily) anti-clockwise around the ring, there are enhanced emotions, a removal of inhibitions, greater intimacy, greater group cohesion, creating a positively-charged social atmosphere which is dominated by the sound of the music, stopping unwanted thoughts and making people feel cool and tough, which in turn motivates them to act in line with the arousing physical sensations. A powerful cocktail. Of course, it is likely that this process would occur on multiple levels simultaneously as well.

This provides a fuller understanding of the theoretical concepts described in the CAALM Model. This proposed that three interacting processes would occur: (1) a physiological adaptation to loud sound; (2) classical conditioning to the loudness, driven by benefits of loud sound; and (3) resulting from these, an acculturation around loud sound amongst those who are so conditioned. The themes emerging from interviews support the theoretical position; they also add to it. One theoretical component which is clarified by the interview data is the presence of benefits of loud sound. The CAALM Model lists perceived external and internal benefits (relative to the mind of a person) of loud sound and also draws on other perceived benefits from entertainment environments, such as friendship, dancing, alcohol, and drugs. The interview data show that the perceived benefits of loud sound are real and that some go beyond mere perception into real advantages in a loud-noise environment. In particular, these include masking effects on thoughts, which have previously tended to be considered as negative aspects of noise exposure [33]; and the association between listening to loud sounds and a sense of personal toughness and power, which we have suggested may reflect a sense of power from overcoming the natural fear and startle responses that could occur due to loud sound but which are known to be under efferent control [34]. It may be that the exercise of this control provides people with a sense of inner strength and thus a feeling of control and toughness.

### 4.1. Health Promotion and the Social Ecological Model Perspective

We believe that a better understanding of the reasons why those who enjoy leisure noise enjoy it may provide avenues for health promotion. Currently, loud sound is accepted by many people within our society, despite the known impacts on health it causes [1]. How can the understanding developed here assist in our consideration of health promotion?

In terms of intervention, a commonly used model for health promotion is the Social Ecological Model. The interview themes can also be aligned with the Social Ecological Model’s levels of influence (Policy, Cultural, Interpersonal, and Intrapersonal). Given that the Policy level already exists for nightclub staff in the form of laws about exposure to high-level sound in the workplace, it is interesting that no mention of this was made in the interviews. It may reflect the principle that health interventions aimed at a single level of the model are ineffective [23], or might suggest that nightclub staff are themselves acculturated to loud music and thus do not consider the sound levels to be a danger. In keeping with these speculations are the findings that there appears to be little effect on exposure levels in nightclubs in places where greater policy controls have been implemented [35,36].

#### 4.1.1. Cultural-Level Influences

At the Cultural level, the main influences appear to be the expectation of loudness from both nightclub staff and clubbers. Nightclub staff used the loud music to influence the customers and clubbers accepted it, even though some felt the levels were too high at times.

Nightclub sound measurements reached levels of around 97 dBA Leq, consistent with those presented elsewhere in recent literature [3]. This level was the maximum level attained during the night, and levels appeared to rise gradually through the course of the night from approximately 85 dBA Leq at 9:15 p.m. up to approximately 97 dBA Leq around midnight and plateauing there (Figure 1). The finding was consistent with our interview findings that there may be a culture or accepted practice of increasing music levels through the course of an evening. The auditory system is highly adaptive to sound levels. In high-level sound, adaptation may occur at multiple sites in the cochlea [37,38,39] and throughout the auditory nervous system up to the level of the cortex [40]. Perceived loudness would depend on both the external level of sound and the degree of physiological adaptation present. Club managers’ auditory systems would adapt to the levels of music through the course of the evening, so maintaining a culturally-accepted level of loudness would require that the sound level be increased as observed. 

The desire to keep sound levels high would be driven by club managers’ desire to meet what they perceive to be the wishes of customers. Our finding that people can find music too loud appears to support other research showing that the real feelings are more mixed [17,18,19], and this would be sensible for managers to consider. Previous research suggests that introducing more policy-level intervention to control the sound levels in the entertainment industry has limited success [35,36], but the consideration that others might not always enjoy the loudness could have more impact on nightclub managers who may be motivated not to drive away potential customers.

#### 4.1.2. Interpersonal-Level Influences

Loud music may draw people together [29]. This may simply reflect the shared experience where the sound of the music dominates the environment so everybody in the room is experiencing a very similar state and the group are all in a joyful, excited mood because of it. Furthermore, people can see that other people are influenced in a similar way due to rhythmic movements that occur as a result of the sound [41].

On the other hand, loud music also allowed people to interact more intimately. In a crowded room, the sound levels would prevent conversations from being overheard, and nightclubs are generally dimly-lit so would allow a degree of privacy. Adding to these factors are the physical proximity required to communicate which may break down social barriers to intimate contact. In other words, the interpersonal effects of loud sound appear mostly to be perceived as benefits by the people who enjoy it. Interventions aimed at this level may therefore be dismissed as irrelevant by the people who would be targeted by them. However, one theme did reflect the difficulties in conversing in noisy environments in pubs, suggesting that in these environments, loud music is not as tolerated. This may provide an avenue to address the spread of loud music beyond the dance club environment.

#### 4.1.3. Intrapersonal-Level Influences

The interview reports of loud music as exciting and arousing reflect an internal factor motivating people to enjoy loud music [28]. Loud music would stimulate people via brainstem mechanisms [42]. The brainstem pathways and nuclei which process sound connect to the reticular formation, which modulates our experience of sound, and is also involved in other sensory systems, initiation and control of motor activity, autonomic arousal, sleep and wakefulness, and emotions [43]. Activation of the reticular formation would occur via loud music, dance, darkness and bright lights, and emotionally-laden social situations and may be expected to contribute to pleasurably heightened arousal.

Music was reported to block out unwanted sound and to provide a more interesting sonic environment. In line with this, personal audio systems have been reported to provide a more personal sound environment [44]. By choosing the sound to which one is exposed, one escapes the tyranny of others controlling one’s environment [45,46]. Furthermore, our data suggest that a listener who enjoys the music and allows themselves to be captured by it may experience a new and better environment, in line with previous theory [29]. Loud music was also reported to mask difficult or troubling thoughts, allowing escape from daily concern.

A sense of identity as a tough and cool person was provided by loud sounds. As mentioned above, the control over loud sound gives one power over oneself and other people [45]. Therefore, the choice of what you yourself listen to and your ability to impose your choices on others would be consistent with the idea of a cool person as someone in control. Furthermore, by mastering one’s own fear-like responses, this sense of toughness may provide reinforcement for loud music due to internal psychophysiology, as described above. It is difficult to see how this would provide an avenue for intervention, but it may explain why loud music is often associated with ‘macho’ or manly personas in our society. Possibly explanation of the postulated mechanism underlying this would, in itself, reduce the sense of misplaced strength and confidence that people obtain.

## 5. Conclusions

The enjoyment of loud sound appears to depend on a complex and powerful interaction of forces (Figure 2). These forces would drive people towards loud music due to cultural, interpersonal and intrapersonal factors, and would thus be difficult to overcome with simple legislative change or well-meaning advice from health professionals. The Social Ecological Model predicts little change in health behaviour without intervention on multiple levels. Laws and regulations against noise exposure at work are ignored in a cultural, interpersonal, and intrapersonal ecology of acceptance of high-level sound. On this basis, interventions would need to address people within a deeper social context.

A preliminary phase of research was conducted as a check that the sound exposures in Auckland were equivalent to those measured in other cities [3]. We found that they were, but incidentally observed the interesting trend of increasing sound through the course of the early evening. It is unclear from our non-systematic study whether this observation is widespread, however it is consistent with the CAALM Model’s adaptation process and with interview data about club policies in regard to sound levels. Future research is desirable to provide more systematically gathered data around this.

Interventions towards safer hearing behaviour have been shown to be effective in young children [47,48], but in teenagers are less effective [47] or non-significant [49]. Effects on teenagers were sustained only when interventions involved them more deeply in the process [50,51]. This is consistent with the power of the influences described here. One approach suggested by this research may be to intervene via the staff of nightclubs: if managers knew that some of the patrons found music too loud, their desire to satisfy their customers might lead them to control sound levels with ideas such as ‘ear rest areas’ and ‘no access areas’ around speakers to help people enjoy loud music more safely and with choice [6]. Furthermore, nightclub staff may be regarded as arbiters of the culture and thus have influence over those who come to the clubs: since the staff are damaging their own hearing by setting music levels so high, there may be opportunities to intervene with them. This research has provided some insight into why people enjoy loud sound building on previous findings [17,18,19], and the theory we have developed around it complements the CAALM Model that we developed [28]. Future research investigating how to break the complex of factors influencing people towards loud sound is important.
